# Diagnosis in Fang Operations

**Published:** 1868-06

**Authors:** Benj. F. Cosby


### ARTICLE II.

### *Diagnosis in Fang Operations.*

By Benj. F. Cosby, D. D. S.

The operation of filling any tooth, in which there is a reasonable doubt as to ultimate success, is one, independent of its immediate difficulties, fraught with more cause for anxiety to the Dental Surgeon, than all his other professional duties combined.

In order to approach an operation of this class, much judgement is required, predicated upon both our experience and that of others. All the requirements of the case, its importance, not only in our own estimation, but in that of the patient, must be fully canvassed before we can fairly decide between the attempt at saving such teeth and that of at once sacrificing them.

Although from a period quite remote, operations of this class have been performed with various degrees of success, the subject has not, until within a comparatively recent period, received that consideration from the profession which it so fully merits. And even at this advanced and enlightened day of our science, many serious difficulties present themselves, and must continue to do so, that have the effect to limit our practice in this important branch.

However successful the most favored of our arts have been, still they have some failures to record.

Without stopping to consider the liability of any oversight as to the thoroughness of the operations performed in those cases, there are other circumstances which should be duly investigated, that must not only govern us first in operating at all, and secondly how far we shall treat the case after filling—if inflammation or alveolar abscess shall supervene—before we should be induced to resort to the radical cure of sacrificing the tooth to the forceps.

The first and great consideration should be as to the importance or probable usefulness of the tooth in question. In forming a diagnosis as to the probable chances of ultimate success, the constitutional indications should be well considered, and should they prove sufficiently satisfactory the liability to continued caries or denudation from causes peculiar to the patient, should not escape our observation.

I shall next consider what condition of decay will provide us with the faintest chances of ultimate success after filling. In regard to class, it is undoubtedly in favor of those teeth that have the least number of fangs, both from this fact, and their natural position being more accessible than the molars. It is therefore proper to presume that incisors and cuspidati would possess advantages over bicuspids, then again over inferior molars, and the last mentioned, over those of the superior jaw, every thing being equal. What I mean by condition of decay is simply this, whether the tooth be already what is commonly designated as "dead," or we are compelled to destroy its internal vitality by arsenic or otherwise. As far as my own experience goes, which has been quite extensive, the prospect of success is much greater in those cases in which we destroy the nerves, than when nature has performed that operation for us, unless we allow too long a period of time to intervene between the filling and the arsenious treatment. In this case it will readily assume all the characteristics of those cases in which a voluntary destruction had ensued. Having said this much in regard to the pathological conditions, and the considerations which must guide us in forming a diagnosis, without offering in detail the treatment to be pursued in operations of this class, I shall only state that prior to filling the fang, or fangs of any tooth, my experience has been, that the greater success will be secured in those cases in which the treatment has been most protracted.

